# Focus on fragility fractures of the pelvis

**DOI:** 10.1007/s00068-020-01550-7

**Published:** 2021-02-01

**Authors:** Pol Maria Rommens, Alexander Hofmann

**Affiliations:** 1 Department of Orthopaedics and Traumatology, University Medical Center of Johannes Gutenberg-University Mainz, Mainz, Germany; 2 Dept. for Traumatology and Orthopaedics, Westpfalz Klinikum Gmbh, Kaiserslautern, Germany

Fragility fractures of the pelvis (FFP) are a clinical entity with an increasing frequency. Epidemiological publications indicate an important growth of the number of female patients with FFP, especially those above the age of 80 [1–3]. FFP are related to a considerable decrease in bone mineral density in the pelvic bone. This phenomenon is responsible for a reduction of its resistance against external forces. The fragility fractures may be localized in the anterior pelvic ring, in the posterior pelvic ring or in both. Patients with an FFP present with intense pain, loss of mobility, and diminution of independency. A comprehensive classification provides a framework for the evaluation of the degree of instability. It differentiates into four main categories: anterior pelvic fractures only, non-displaced posterior fractures, and unilateral resp. bilateral displaced posterior fractures. The classification also provides recommendations for the type of treatment needed [4, 5]. Nevertheless, there is limited evidence about origination, natural progress, and the most appropriate treatment of FFP until today. The frailty of these elderly patients, which suffer FFP, requires specific, less invasive treatment algorithms.

In this focus, five groups of investigators shed light on different aspects of FFP, which not have been recognized so far. They all add to the existing knowledge and indicate further clinical and biomechanical investigation. Lee et al. from Korea analyzed the possible correlation between pelvic morphology and occurrence of FFP. They distinguished between a circle type—with an equal transverse and sagittal true pelvis diameter—and an ellipse type with a larger transverse true pelvis diameter. The authors found that there was a 4.1 higher risk of suffering an FFP in patients with a circle type true pelvis in their patient cohort. This matter of fact may be related to the specific distribution of forces, which impact laterally on the pelvis during a fall on the side [6].

In their retrospective analysis, Mendel et al. from Germany observed the sequence of fracture progression in sacral fragility fractures. A unilateral sacral fracture is followed by a contralateral one. Bilateral sacral fractures are subsequently interconnected by a horizontal fracture line at the level of S1 or S2. Most interestingly, increasing instability occurs through the onset of iliolumbar ligament avulsion. Based on the analysis of diagnostic images, an indirect conclusion can be drawn about the duration of the suffering [7]. These data put the observations of Linstrom et al. from 2009 in a dynamic perspective [8].

Two publications from Japan—the country with one of the oldest populations in the world – focus on the feasibility and outcome of conservative treatment of FFP. Hotta et al. conducted a retrospective study on 84 patients. Functional treatment was performed in all patients, who could be mobilized within pain limits in the first 10 days after trauma. The authors did not find a difference in functional outcome between patients with FFP Type II and patients with FFP Type III–IV. Although a fracture progression was found in nearly one-fifth of the patients with FFP Type II, surgical intervention was only performed in one-tenth. Time until the bony union was longer than 3 months in FFP Type II and five months in FFP Type III–IV. The authors concluded that outcome after conservative management is not dependent on FFP-fracture classification [9].

Yosida et al. retrospectively reviewed 340 patients with FFP treated in one single trauma unit. Surgical treatment was performed in 16 (4.7%) patients only. Only one-third of the patients, who were treated conservatively and could be followed for more than 1 year, regained their standing and walking abilities, whilst more than two-thirds of such patients were detected in the surgical group. The authors concluded that FFP greatly affects activities of daily living and that surgical treatment may be needed to reliably regain standing and walking ability [10]. Although surgical treatment may be beneficial, there still is much reluctance due to the invasiveness of surgical procedures and their possible negative impact on elderly persons. There is a tendency for less invasive surgery with the goal of restoring stability rather than anatomy [11].

Schmerwitz et al. from Germany evaluated the usefulness of locked compression plates, which are inserted minimally invasive, for fixation of the broken posterior pelvis. They reviewed the medical data of 53 patients retrospectively. There were surgery-related complications in 13% of patients. 79.2% returned to their previous living situation after a median hospital stay of 21 days. The authors concluded that minimal-invasive stabilization of the fractured posterior pelvis with a locked compression plate may be a beneficial treatment for elderly patients with FFP [12].

Literature data and clinical evidence on origin, natural course, treatment alternatives, and outcome of patients with FFP are increasing. With this focus, we want to enhance the already existing knowledge and add new aspects and data, which may help the reader in its own clinical environment choosing the best options for these fragile patients. We wish you interesting and instructive readings.

## References

[1] Sullivan MP, Baldwin KD, Donegan DJ, Mehta S, Ahn J (2014). Geriatric fractures about the hip: divergent patterns in the proximal femur, acetabulum, and pelvis. Orthopedics.

[2] Andrich S, Haastert B, Neuhaus E, Neidert K, Arend W, Ohmann C, Grebe J, Vogt A, Jungbluth P, Rösler G, Windolf J, Icks A (2015). Epidemiology of pelvic fractures in Germany: considerably high incidence rates among older people. PLoS ONE.

[3] Kannus P, Parkkari J, Niemi S, Sievänen H (2015). Low-trauma pelvic fractures in elderly finns in 1970–2013. Calcif Tissue Int.

[4] Rommens PM, Hofmann A (2013). Comprehensive classification of fragility fractures of the pelvic ring: recommendations for surgical treatment. Injury.

[5] Rommens PM, Wagner D, Hofmann A (2017). Fragility fractures of the pelvis. JBJS Rev.

[6] Lee HH, Kim WY, Lim YW, Byun YS, Lee SW (2020). Is there a correlation between fragility fractures of the pelvis (FFP) and the morphology of the true pelvis in geriatric patients?. Eur J Trauma EmergSurg.

[7] Mendel T, Ullrich BW, Hofmann GO, Schenk P, Goehre F, Schwan S, Klauke F (2020). Progressive instability of bilateral sacral fragility fractures in osteoporotic bone: a retrospective analysis of X-ray, CT, and MRI datasets from 78 cases. Eur J Trauma EmergSurg.

[8] Linstrom NJ, Heiserman JE, Kortman KE, Crawford NR, Baek S, Anderson RL, Pitt AM, Karis JP, Ross JS, Lekovic GP (2009). Anatomical and biomechanical analyses of the unique and consistent locations of sacral insufficiency fractures. Spine (Phila Pa 1976).

[9] Hotta K, Kobayashi T (2020). Functional treatment strategy for fragility fractures of the pelvis in geriatric patients. Eur J Trauma EmergSurg.

[10] Yoshida M, Tajima K, Saito Y, Sato K, Uenishi N, Iwata M (2020). Mobility and mortality of 340 patients with fragility fracture of the pelvis. Eur J Trauma EmergSurg.

[11] Rommens PM (2019). Paradigm shift in geriatric fracture treatment. Eur J Trauma EmergSurg.

[12] Schmerwitz IU, Jungebluth P, Lehmann W, Hockertz TJ (2020). Minimally invasive posterior locked compression plate osteosynthesis shows excellent results in elderly patients with fragility fractures of the pelvis. Eur J Trauma Emerg Surg..

